# Additive Pharmacological Interaction between Cisplatin (CDDP) and Histone Deacetylase Inhibitors (HDIs) in MDA-MB-231 Triple Negative Breast Cancer (TNBC) Cells with Altered Notch1 Activity—An Isobolographic Analysis

**DOI:** 10.3390/ijms20153663

**Published:** 2019-07-26

**Authors:** Anna Wawruszak, Jarogniew J. Luszczki, Joanna Kalafut, Karolina Okla, Marta Halasa, Adolfo Rivero-Muller, Andrzej Stepulak

**Affiliations:** 1 Department of Biochemistry and Molecular Biology, Medical University of Lublin, Chodzki 1 Street, 20-093 Lublin, Poland; 2 Isobolographic Analysis Laboratory, Institute of Rural Health, Jaczewskiego 2 Street, 20-090 Lublin, Poland; 3 Department of Pathophysiology, Medical University, Jaczewskiego 8 Street, 20-090 Lublin, Poland; 4 The First Department of Gynecologic Oncology and Gynecology, Medical University of Lublin, Staszica 16 Street, 20-081 Lublin, Poland; 5 Tumor Immunology Laboratory, Medical University of Lublin, Staszica 16 Street, 20-081 Lublin, Poland; 6 Faculty of Science and Engineering, Cell Biology, Abo Akademi University, Tykistokatu 6A, 20520 Turku, Finland

**Keywords:** triple negative breast cancer (TNBC), cisplatin (CDDP), histone deacetylase inhibitors (HDIs), valproic acid (VPA), vorinostat (SAHA), isobolographic analysis, Notch1 receptor

## Abstract

The aim of this study was to investigate the influence of the Notch1 activity level on the pharmacological interaction between cisplatin (CDDP) and two histone deacetylase inhibitors (HDIs)—valproic acid (VPA) and vorinostat (SAHA) in the triple negative breast cancer (TNBC) cells. Stable breast cancer (BC) cell lines with increased and decreased activity of Notch1 were generated using a transfection method. The type of interaction between CDDP and the HDIs was determined by isobolographic analysis of cell proliferation in MDA-MB-231 cells with differential levels of Notch1 activity in vitro. The combination of CDDP/SAHA and CDDP/VPA in the MDA-MB-231 triple negative breast cancer (TNBC) cells with increased activity of Notch1, as well as CDDP/VPA in the MDA-MB-231 cells with decreased activity of Notch1, yielded an additive interaction, whereas additivity with a tendency towards antagonism was observed for the combination of CDDP/SAHA in MDA-MB-231 cells with the decreased activity of Notch1. Our studies demonstrated that SAHA and VPA might be considered as potential therapeutic agents in combination therapy with CDDP against TNBC with altered Notch1 activity.

## 1. Introduction

Breast cancer (BC) is the most common cancer diagnosed among women worldwide. A recent global cancer report released by the International Agency for Research on Cancer (IARC) revealed that BC accounts for 25% of all types of cancer cases, and 15% of all malignancy deaths among women globally [1,2]. 

Based on molecular profiling and the presence of estrogen (ER), progesterone (PR), and HER2 receptors, as well as the intensity of Ki-67 protein expression, breast cancers are categorized into five principal molecular subtypes: Luminal A ([ER+/PR+] HER2-Ki67−), luminal B ([ER+/PR+] HER2-KI67+) or ([ER+/PR+] HER2+KI67+), HER2 over-expression ([ER−/PR−] HER2+), basal ([ER−/PR−] HER2−, basal marker+), and normal-like ([ER+|PR+] HER2-KI67−), which shares a similar immunohistochemical (IHC) status with the luminal A subtype, but is characterized by normal breast tissue profiling [3]. 

Most basal-like BCs are qualified as a triple negative breast cancer (TNBC). TNBC accounts for 10%–20% of all BCs and correlates with poor outcomes, with a high rate of local and systemic relapse. Since TNBC lacks ER, PR, and HER2 receptors, they do not respond to targeted treatment agents, such as trastuzumab or tamoxifen [4,5]. Chemotherapy options for women with TNBC are only managed with standard chemotherapy, such as paclitaxel [4] or platinum-based compounds [6].

**Figure 1:**
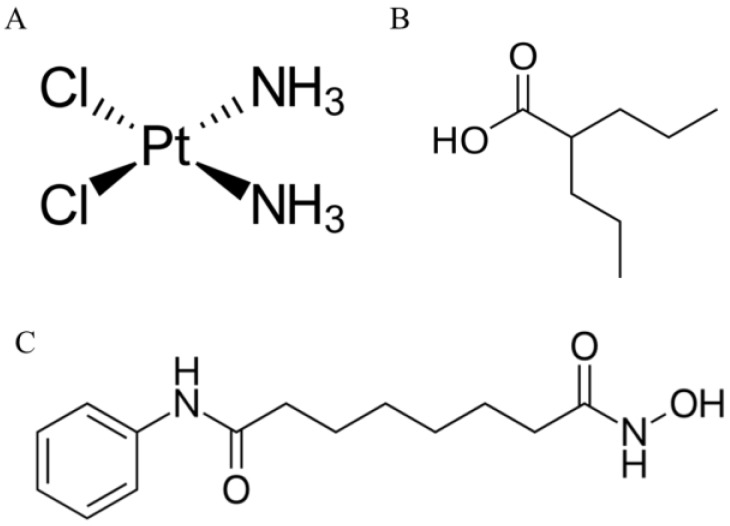
Chemical structures of (**A**) cisplatin (cis-diamminedichloroplatinum (II), cisplatin (CDDP)); (**B**) valproic acid (VPA) and (**C**) vorinostat (suberoylanilide hydroxamic acid, SAHA).

Cisplatin (cis-diamminedichloroplatinum (II), CDDP) (Figure 1A) [7] is a DNA-damaging chemotherapy agent used in the therapy of many types of cancer [8,9,10], including TNBC [11,12,13]. CDDP inhibits cell proliferation through binding with DNA to create intra-strand adducts changing DNA conformation [14], favoring mitochondrial damage, altering cellular transport mechanisms, and reducing ATPase activity inside the cells [15,16]. Unfortunately, CDDP and other standard chemotherapeutic agents cause serious adverse side effects, such as neurotoxicity [17], myelosuppression, or gastrointestinal toxicity [18]. The use of CDDP is hindered by CDDP-resistance development, which can partially be overcome by the use of combined therapy. This type of therapy not only improves the efficacy of drugs used separately, but also lowers the doses of chemotherapeutic agents, which can lead to a decrease in the adverse effects, delays tumor recurrence, and results in an increase in the quality of life of patients [19].

A noticeable contribution of epigenetic changes to the development and maintenance of BC has been underlined [20,21]. In this context, a new class of antineoplastic drugs, affecting histone acetylation, has been introduced for cancer therapy [22,23]. HDIs have been evaluated in breast cancer in vitro and in vivo, as single agents or in combined therapy, providing promising results [24]. Vorinostat (suberoylanilide hydroxamic acid, SAHA) (Figure 1C) is a powerful active agent targeting most histone deacetylases (HDAC) classes I and II. An additional advantage of SAHA is its high bioavailability [25], and the ability to cross the blood–brain barrier preventing the formation of brain metastases [24]. Valproic acid (VPA) (Figure 1B) is a short-chain fatty acid that has been commonly used in the therapy of epilepsy and other neuropsychiatric disorders for the last two decades. VPA was also designated as one of the potent HDIs with effective anticancer activity [26]. Similarly to SAHA, VPA can suppress the growth of BC cells by inducing apoptosis and arresting them in the G1 phase [27].

In our previous study, sub-additive (antagonistic) interaction was observed for the combination of CDDP with VPA in MDA-MB-231 TNBC cells, whereas a combination of CDDP with SAHA in the same MDA-MB-231 cell line yielded additive interaction [28]. Since it is known that VPA affects Notch1 activity [29], this discrepancy led us to examine if reduction or over expression of Notch receptor activity will affect the drug–drug interaction in combined treatment. 

Notch is a trans-membrane receptor that plays roles as either a tumor suppressor or an oncogene depending on the molecular context [30]. Notch signaling plays essential roles in maintaining the balance between cell proliferation, differentiation, and apoptosis [31]. In humans, there are four types of Notch receptors (Notch1-Notch 4) and five Delta, Serrate, Lag2 (DSL) ligands (Jagged1, Jagged2, Delta-like1 (Dll1), Delta-like3 (Dll3), and Delta-like4 (Dll4)). Notch signaling is initiated by the interplay of DSL ligands and Notch receptors on conterminous cells, where mechanical pulling of the receptor’s extracellular domain results in the exposure of a cryptic protease site, triggering a cascade of proteolytic cleavages, culminating in the release of Notch intracellular domain (NICD), which then translocates to the nucleus and interacts with the DNA binding protein CBF1/Su(H)/Lag-1 (CSL) (aka recombination signal binding protein for immunoglobulin kappa (RBP-Jk) or centromere-binding protein 1 (CBF1)) [32]. In the ‘‘Notch off’’ state, CSL acts as a repressor and binds other transcriptional co-repressors, such as lysine-specific demethylase 5A (KDM5A), SMART/HDAC1-associated repressor protein (SHARP), and KyoT2. In turn, in the ‘‘Notch on’’ state, the NICD/CSL complex recruits co-activators, such as p300 and the acetyltransferase p300/CBP associated factor (PCAF), becoming a transcriptional activator [33]. 

It has been noted that four Notch paralogs play different roles in the development of BC. A high level of Notch1, Notch3, and Notch4 expression is associated with poor clinical outcomes of BC, in contrast to Notch2, which was recognized as a neoplasm suppressor [34]. The exact significance of different isoforms of Notch in BC is unclear, however, Notch1 seems to be vital for BC progression [35]. It has also been reported that an increase in the expression of Notch1 correlates with a dramatic reduction of the overall survival of BC patients. Notch1 is related to almost every stage of BC, such as ductal carcinomas in situ (DCISs), infiltrating ductal carcinomas (IDCs), and infiltrating lobular carcinomas (ILCs) [35]. 

It has been demonstrated that VPA affects Notch-mediated signaling [29,36,37]. However, no data is available on whether Notch activity has an impact on the success or failure of received treatment in patients with BC. Therefore, the aim of the present study was to assess the influence of dysregulated Notch1 activity for HDIs and CDDP mediated inhibition of TNBC cancer cell proliferation.

## 2. Results

### 2.1. Expression and Activity of Notch1

**Figure 2:**
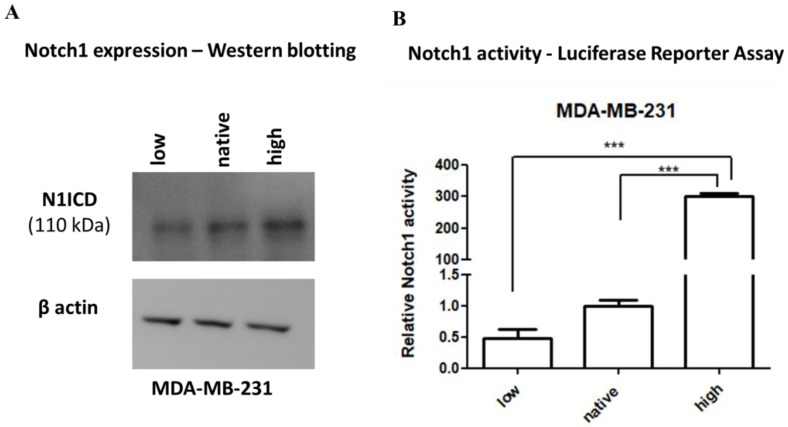
Immunoblotting and Luciferase Reporter Assay of Notch1^high^ (N1ICD), Notch1^low^ (dnCSL), or native MDA-MB-231 breast cancer cells. (**A**) MDA-MB-231 breast cancer cells transfected with ΔEN1ICD or dnCSL show that ΔEN1ICD increases the levels of N1ICD protein, while no changes were detected in dnCSL as compared to native MDA-MB-231 cells. Anti-β-actin antibody was used as a control for equal loading. Representative blots from tree-independent experiments are shown; (**B**) Notch signaling activity from the 12xCSL-luc reporter in MDA-MB-231, Notch1^low^MDA-MB-231, and Notch1^high^MDA-MB-231 breast cancer cells. The relative activity of Notch1 was normalized to the activity of native MDA-MB-231 cells. The results are presented as mean ± standard error of mean (±S.E.M). Statistical analysis was performed using a one-way ANOVA test, Tukey post-hoc testing (*** *p* < 0.001).

Two MDA-MB-231 cell lines were created, one having high (Notch1^high^MDA-MB-231) and the other low (Notch1^low^MDA-MB-231) Notch1 activity. This was achieved by stably transfecting either the ΔEN1ICD, a truncated Notch1 that is immediately cleaved at the membrane releasing N1ICD [38], or the dominant negative CSL (dnCSL), a cytoplasmic CSL that sequesters any active NICD before it can translocate to the nucleus [38], respectively. Notch1^high^MDA-MB-231 expressed much higher levels of NICD, while the Notch1^low^MDA-MB-231 line has a similar NICD level at wild type (WT) parental MDA-MB-231 cells (native), as shown by immunoblotting (Figure 2A). Notch1^high^MDA-MB-231 cells had high Notch activity, as analyzed by a reporter assay, while Notch1^low^MDA-MB-231 had lower Notch1 activity than native cells (Figure 2B). 

### 2.2. Assessment of Notch1 Gene Expression Changes after HDIs and CDDP Treatment

**Figure 3:**
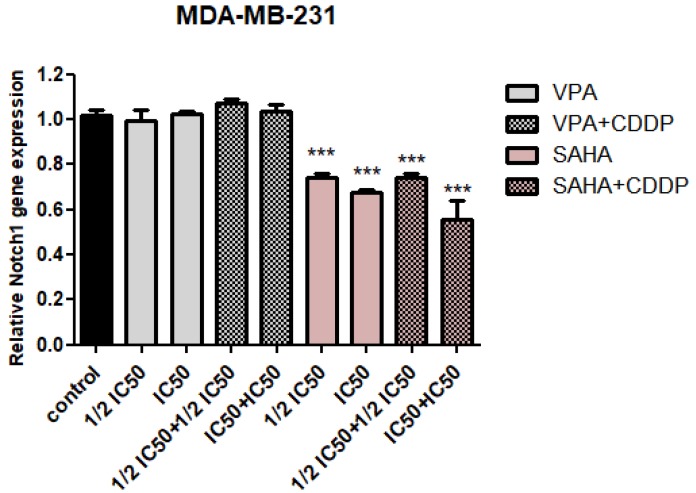
The mRNA expression level of Notch1 in MDA-MB-231 breast cancer cells after (histone deacetylase inhibitors) HDIs and CDDP treatment. Expression of Notch1 was analyzed by qPCR in MDA-MB-231 cells exposed to the culture medium alone (control), VPA (½ IC_50_; IC_50_), or SAHA (½ IC_50_; IC_50_) individually or in combination with CDDP (½ IC_50_ + ½ IC_50_, IC_50_ + IC_50_) for 24h. The differences between groups were evaluated using the one-way analysis of variance (ANOVA); Tukey’s post-hoc test. *p* < 0.05 was considered to indicate a statistically significant difference (*** *p* < 0.001). Results from three independent experiments (*n* = 9) were presented as the mean ± standard error of the mean (±S.E.M).

qPCR analysis revealed that SAHA significantly decreased of Notch1 gene expression in a dose-dependent manner. A similar tendency was observed for the combination of SAHA and CDDP. In the case of the IC_50_ SAHA + IC_50_ CDDP combination, a nearly 40% decrease in Notch1 expression level was observed. There were no statistically significant differences in Notch1 expression between control and VPA treatment individually, or in combination with cisplatin, at the mRNA level, as seen by the qPCR method (Figure 3). 

### 2.3. Dose-Dependent Growth Inhibition of Native and Transfected MDA-MB-231 Breast Cancer Cells after CDDP and HDIs Treatment

**Figure 4:**
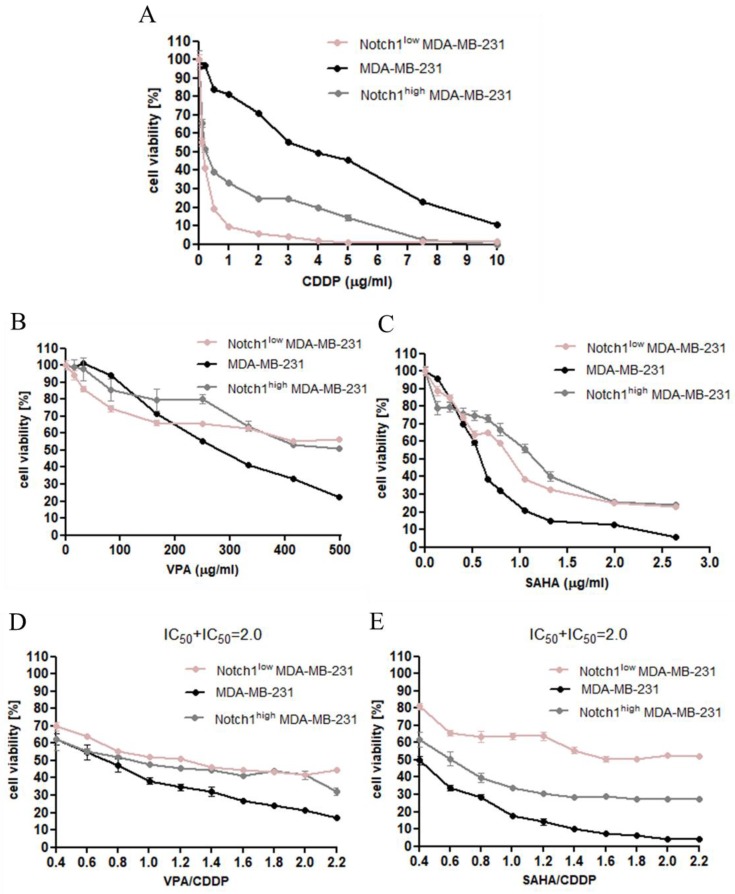
The anti-proliferative effects of CDDP and HDIs in MDA-MB-231 breast cancer cells. (**A**) The anti-proliferative effect of CDDP in MDA-MB-231 [28], Notch1^low^MDA-MB-231, and Notch1^high^MDA-MB-231 breast cancer cells; (**B**) the anti-proliferative effect of VPA in MDA-MB-231 [28], Notch1^low^MDA-MB-231, and Notch1^high^MDA-MB-231 breast cancer cells; (**C**) the anti-proliferative effect of SAHA in MDA-MB-231 [28], Notch1^low^MDA-MB-231, Notch1^high^MDA-MB-231 breast cancer cells; (**D**) the anti-proliferative effect of combined treatment of VPA and CDDP in MDA-MB-231 [28], Notch1^low^MDA-MB-231, and Notch1^high^MDA-MB-231 breast cancer cells; (**E**) the anti-proliferative effect of combined treatment of SAHA and CDDP in MDA-MB-231 [28], Notch1^low^MDA-MB-231, and Notch1^high^MDA-MB-231 breast cancer cells. Transfected and native MDA-MB-231 cells were exposed to concomitant HDIs and CDDP treatment using different ratios of the IC_50_ (2.0 = IC_50_ + IC_50_). The cell viability was measured by the MTT assay. The results from three independent experiments (*n* = 18) are presented as the mean ± standard error of the mean (±S.E.M).

The cytotoxic effect of CDDP, VPA, and SAHA was determined in the MDA-MB-231 breast cancer cell lines with increased and decreased Notch1 activity using the 3-(4,5-dimethylthiazol-2-yl)-2,5-diphenyltetrazolium bromide (MTT) assay in order to establish the IC_50_ value for each analyzed compound in all cell lines (Table 1). In our study, we have demonstrated the dose-dependent growth inhibition effect of each compound in all analyzed breast cancer cell lines. As shown in Figure 4A, the cytotoxic effect of CDDP was higher for MDA-MB-231 cells with altered Notch1 activity than native breast cancer cells. A similar tendency was only observed when low concentrations of VPA (up to 150 µg/mL) and SAHA (up to 0.5 µg/mL) were used. At higher doses of HDIs, the transfected cells were more resistant to the VPA and SAHA than native MDA-MB-231 cells (Figure 4B,C). Next, we focused on the growth inhibition effect of a combination of CDDP with HDIs. In both cases, untransfected breast cancer cells treated with a combination of CDDP with VPA and CDDP with SAHA were much more sensitive than cells with altered Notch1 activity (Figure 4D,E).

### 2.4. Effect of SAHA and VPA on The Anti-Proliferative Effects of CDDP in The MDA-MB-231 Cell Line with Increased Activity of The Notch 1 (Notch1^high^MDA-MB-231)

**Figure 5:**
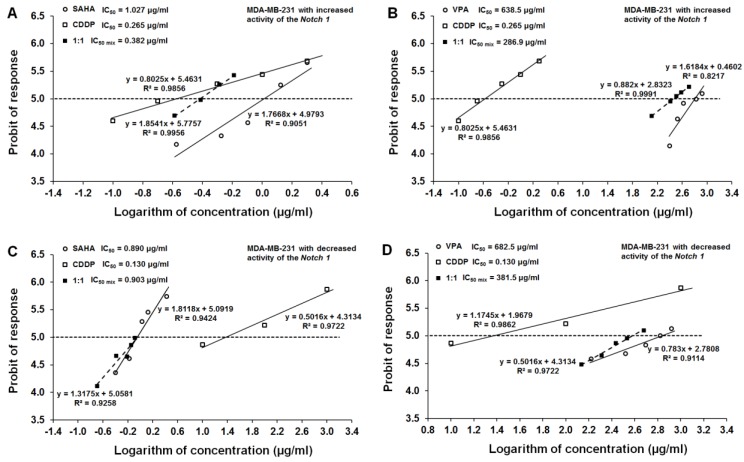
Log-probit dose-response relationship lines for HDIs and CDDP in transfected MDA-MB-231 cells. (**A**) Log-probit dose-response relationship lines for CDDP and VPA administered alone, and in combination at the fixed-ratio of 1:1, with respect to their anti-proliferative effects on the cancer cell lines MDA-MB-231 with increased activity of Notch1 (Notch1^high^MDA-MB-231); (**B**) Log-probit dose-response relationship lines for CDDP and SAHA administered alone, and in combination at the fixed-ratio of 1:1, with respect to their anti-proliferative effects on the cancer cell lines MDA-MB-231 with increased activity of Notch1 (Notch1^high^MDA-MB-231); (**C**) Log-probit dose-response relationship lines for CDDP and VPA administered alone, and in combination at the fixed-ratio of 1:1, with respect to their anti-proliferative effects on the cancer cell lines MDA-MB-231 with decreased activity of Notch1 (Notch1^low^MDA-MB-231); (**D**) Log-probit dose-response relationship lines for CDDP and SAHA administered alone, and in combination at the fixed-ratio of 1:1, with respect to their anti-proliferative effects on the cancer cell lines MDA-MB-231 with the decreased activity of Notch1 (Notch1^low^MDA-MB-231). Doses of particular compounds (CDDP, SAHA, and VPA) administered both separately and in combination were transformed into logarithms, whereas the anti-proliferative effects produced by the drugs in the cancer cell line MDA-MB-231 were transformed into probits according to [39]. Equations of dose-response relationship lines are presented on the multipart figure. Respective IC_50_ values are depicted in the left corners in each part of the figure.

All three tested compounds, CDDP, SAHA, and VPA, exerted a clear-cut anti-proliferative effect on the MDA-MB-231 cell line with increased activity of the Notch1 (Figure 4). Log-probit dose-response effects allowed for the calculation of the IC_50_ values for CDDP, SAHA, and VPA which were 0.265 ± 0.196 μg/mL, 1.027 ± 0.386 μg/mL, and 638.5 ± 234.3 μg/mL, respectively (Figure 5A,B). Additionally, all dose-response effect (log-probit) lines between CDDP and SAHA and CDDP and VPA for the MDA-MB-231 cell line with increased activity of the Notch1 were not parallel to one another (Figure 5A,B). 

### 2.5. Effect of SAHA and VPA on The Anti-Proliferative Effects of CDDP in The MDA-MB-231 Cell Line with Decreased Activity of The Notch 1 (Notch1^low^MDA-MB-231)

The single administration of CDDP, SAHA, and VPA resulted in a clear-cut anti-proliferative effect on the MDA-MB-231 cell line with decreased activity of the Notch1 (Figure 4). In this cancer cell line, the IC_50_ values for CDDP, SAHA, and VPA were 0.130 ± 0.060 μg/mL, 0.890 ± 0.292 μg/mL, and 682.5 ± 517.6 μg/mL, respectively (Figure 5C,D). All dose-response effect (log-probit) lines between CDDP and SAHA and CDDP and VPA for the MDA-MB-231 cell line with decreased activity of the Notch1 were not parallel to one another (Figure 5C,D).

### 2.6. Type I Isobolographic Analysis of Interaction for The Combinations of CDDP with SAHA and VPA in The MDA-MB-231 Cell Line with Increased Activity of The Notch 1 (Notch1^high^MDA-MB-231)

**Figure 6:**
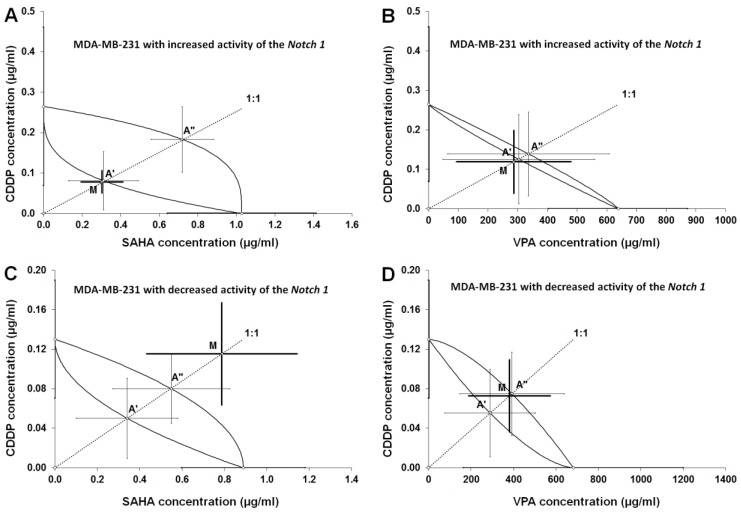
Isobolograms illustrating additive interactions between CDDP and HDIs in transfected MDA-MB-231 cells. (**A**) Isobologram illustrating additive interactions between CDDP and SAHA with respect to their anti-proliferative effects on the MDA-MB-231 cells with the increased activity of Notch1 (Notch1^high^MDA-MB-231); (**B**) isobologram illustrating additive interactions between CDDP and VPA with respect to their anti-proliferative effects on the MDA-MB-231 cells with increased activity of Notch1 (Notch1^high^MDA-MB-231); (**C**) isobologram illustrating additive interactions between CDDP and SAHA with respect to their anti-proliferative effects on the MDA-MB-231 cells with decreased activity of Notch1 (Notch1^low^MDA-MB-231); (**D**) isobologram illustrating additive interactions between CDDP and VPA with respect to their anti-proliferative effects on the MDA-MB-231 cells with decreased activity of Notch1 (Notch1^low^MDA-MB-231). The IC_50_ ± S.E.M. for CDDP, SAHA, and VPA are plotted graphically on the X- and Y- axes, respectively. The lower and upper isoboles of additivity represent the curves connecting the IC_50_ values for CDDP and SAHA, or VPA administered alone. The points A’ and A” depict the theoretically calculated IC_50_ add values (± S.E.M.) for both, lower and upper isoboles of additivity. The point M on each graph represents the experimentally-derived IC_50_ mix value (± S.E.M.) for the total dose of the mixture, which produced a 50% anti-proliferative effect in the cancer cell line MDA-MB-231. The experimentally-derived IC_50_ mix value is placed close to the point A’ for the lower isobole of additivity (**A**), (**B**), indicating additive interaction between CDDP and SAHA or CDDP and VPA in MDA-MB-231 breast cancer cells with increased expression of Notch1 (Notch1^high^MDA-MB-231). The experimentally-derived IC_50_ mix value is placed above the point A” (**C**) or close to the point A” for the upper isobole of additivity (**D**), indicating an additive interaction between CDDP and VPA, and additive interaction with a tendency towards antagonism between CDDP and SAHA in the MDA-MB-231 cells with the decreased activity of Notch1 (Notch1^low^MDA-MB-231).

The combinations of CDDP with SAHA and CDDP with VPA at the fixed-ratio of 1:1 exhibited the definite anti-proliferative effects in the MDA-MB-231 cell line with the increased activity of the Notch1, and the experimentally-derived IC_50_ mix values for the two-drug mixture were 0.382 ± 0.137 μg/mL (CDDP with SAHA; Table 2, Figure 6A), and 286.9 ± 193.2 μg/mL (CDDP with VPA; Table 2, Figure 6B). Type I isobolographic analysis for non-parallel dose-response effects did not reveal any significant differences between the IC_50_ mix and IC_50_ add values (unpaired Student’s t-test). Thus, the analyzed interactions between CDDP and SAHA or VPA were added (Table 3, Figure 6A,B).

### 2.7. Type I Isobolographic Analysis of Interaction for The Combinations of CDDP with SAHA and VPA in The MDA-MB-231 Cell Line with Decreased Activity of The Notch 1 (Notch1^low^MDA-MB-231)

Likewise, the combinations of CDDP with SAHA and CDDP with VPA (at the fixed-ratio of 1:1) produced the definite anti-proliferative effects in the MDA-MB-231 cell line with the decreased activity of the Notch1. The experimentally determined IC_50_ mix values for the two-drug mixture were 0.903 ± 0.407 μg/mL (CDDP with SAHA; Table 2, Figure 6C), and 381.5 ± 192.9 μg/mL (CDDP with VPA; Table 2, Figure 6D). Type I isobolographic analysis for non-parallel dose-response effects revealed that no significant differences were observed between the IC_50_ mix and IC_50_ add values (unpaired Student’s t-test). A lack of statistically significant difference confirms that the analyzed interaction between CDDP and SAHA was additive, although a slight (non-significant) tendency towards antagonism was observed (Table 3, Figure 6C). The combination of CDDP with VPA in the MDA-MB-231 cell line with decreased activity of the Notch1 produced additivity (Table 3, Figure 6D).

### 2.8. Analysis of The Types of Pharmacological Interaction between CDDP and HDIs in The MDA-MB-231 Breast Cancer Cells with Altered Notch1 Activity with Reference to Native MDA-MB-231 Breast Cancer Cells

Isobolographic analysis of interaction for non-parallel DRRCs revealed that the mixture of CDDP with VPA and CDDP with SAHA at the fixed-ratio of 1:1 exerted an additive interaction in the Notch1^high^MDA-MB-231 cell line. A similar tendency was observed in the Notch1^low^MDA-MB-231 cells co-treated with CDDP and VPA. Additivity with a tendency towards antagonism was observed for only the combination of CDDP/SAHA in MDA-MB-231 cells with decreased activity of Notch1. In addition, a better type of pharmacological interaction between CDDP and VPA has been observed in the cells with altered Notch activity compared to the native cells. Therefore, VPA may be used in the combined therapy with CDDP against a very aggressive type of breast cancer—TNBC with increased Notch1 activity. In the case of Notch1^low^MDA-MB-231 cells, the type of isobolographic interaction depends on the type of HDIs used (CDDP/VPA—additivity, CDDP/SAHA—additivity with a tendency towards antagonism). In summary, our studies demonstrated that SAHA and VPA might be considered as potential therapeutic agents in therapy with CDDP against TNBC with altered Notch1 activity.

## 3. Discussion

During recent years, there has been a renewed interest in platinum compounds in the treatment of triple negative breast cancer (TNBC) patients [40]. However, chemotherapy with cisplatin (CDDP), or its derivatives, is limited due to high toxicity to normal cells, many side effects, low therapeutic index, as well as the occurrence of CDDP tolerance [41]. Combination therapy, using drugs with different mechanisms of action, is often used in cancer treatment to overcome these problems [28]. In this context, a broad range of natural and synthetic chemical compounds, including histone deacetylases inhibitors (HDIs), have been identified and have become an interesting class of agents for tumor therapy [42]. In our recently published studies, we have shown the beneficial effects of combined CDDP/HDIs treatment against TE671 human rhabdomyosarcoma [43], A549, NCI-H1563 human adenocarcinoma, NCI-H2170 human squamous cell carcinoma [41], and RK33 human larynx cancer cells [42], utilizing advanced isobolographic analysis of drug-drug interaction. The isobolography is a very rigorous and precise pharmacodynamic method to establish the type of interaction between different active agents, which exhibit a broad range of concentrations. However, this method is not commonly used to determine the types of pharmacological drug–drug interactions in cancer related studies. Instead, simple correlations between tested agents are usually demonstrated, where only a limited number of chosen doses are selected [22]. 

It has been shown that HDIs affect Notch-mediated signaling. Notch signaling in cancer has cell- and context-dependent roles and it can be tumor-suppressive or tumor-stimulating. VPA treatment seems to induce the down-regulation of Notch activity via the suppression of its HES family bHLH transcription factor 1 (HES1) target gene, with augmenting p21 and p63 tumor suppressors in hepatocellular carcinoma cells [37]. Moreover, VPA inhibits cell proliferation of the RPMI 8226 multiple myeloma cells, possibly through the inhibition of the Notch signaling pathway [44]. In contrast, VPA and suberoylbishydroxamic acid (SBHA) effectively upregulate Notch1 activity and suppress neuroendocrine (NE) tumor markers, induced apoptosis, and cell cycle arrest in vitro and in vivo [45,46]. Likewise, it has been shown that treatment of neuroblastoma (NB) [47], human gastrointestinal, and pulmonary carcinoid cancer cells with VPA caused an increase in Notch1 activity and inhibition of cancer cell growth in vitro and in vivo in a mouse xenograft model [48]. VPA suppressed small-cell lung cancer (SCLC) cell growth and caused cell cycle arrest at phase G1, as well as activated Notch signaling by an increase of Notch1, Notch target gene HES1, and p21 expression [49]. Here, we show that SAHA individually or in combination with CDDP significantly reduces the expression of Notch1, which might be beneficial for this particular cancer type where clear oncogenic Notch1 signaling has been described [50,51,52]. 

Isobolographic analysis of the interaction between HDIs and CDDP revealed that the mixture of CDDP with VPA and CDDP with SAHA at the fixed-ratio of 1:1 exerted an additive interaction in the Notch1^high^MDA-MB-231 breast cancer cells. A similar tendency was observed in the Notch1^low^MDA-MB-231 cells co-treated with CDDP and VPA. Therefore, these compounds can be successfully used together in patients with altered (increased or decreased) Notch1 activity. Additivity with a tendency towards antagonism was only observed in the combination of CDDP/SAHA in the Notch1^low^MDA-MB-231 cell line. 

In addition, a better type of pharmacological interaction between CDDP and VPA has been observed in the cells with altered Notch activity compared with the native cells, which we analyzed in our previous paper [28]. Obtained results could be promising, especially for patients with the most aggressive type of BC (TNBC) with a high level of Notch1 activity. 

In the case of Notch1^low^ MDA-MB-231 cells, the type of isobolographic interaction depends on the type of HDIs used (CDDP/VPA—additivity, CDDP/SAHA—additivity with tendency towards antagonism). Both tested HDIs produce epigenetic changes, affecting several genes’ expression by inhibiting selected types of HDACs and increasing histone acetylation [20]. Thereby, they could affect a set of genes differentially expressed in the analyzed cell line, resulting in a varied response for the applied treatment. Differences in the type of CDDP/SAHA interaction in MDA-MB-231 cells with increased and decreased activity of Notch1 (additivity versus additivity with a tendency towards antagonism) seem to be very intriguing. While transfected cells with low and high Notch1 activity represent the same histopathological characteristics, combinatorial use of SAHA with CDDP resulted in a different response. The mechanism underlying this phenomenon is not clear and requires further research. 

Another fact needs special explanation, especially when isobolographically compared the IC_50_ mix values produced by the same two-drug mixture (i.e., CDDP+SAHA), in the same cancer line (i.e., MDA-MB-231), but with different Notch1 activity (Notch1^low^ vs. Notch1^high^). As was illustrated in Figure 6A,C, the two-drug mixture in both cases exerted additivity, but for the increased activity of Notch1, a low dose of two-drug mixture is required to reach the same anti-proliferative effect (i.e., 50% inhibition of proliferation). In contrast, the decreased activity of Notch1 in the cancer cells resulted in additivity with a tendency towards antagonism, which means that a high dose of two-drug mixture is required to reach the same effect (50% inhibition of proliferation). With isobolography, we confirmed that, to inhibit cancer cells, it is obligatory to sometimes arrange the individual dose of a two-drug mixture. In clinical practice, we can observe an identical situation related with better, normal, or worse response to the applied anti-cancer treatment. It seems that, due to the expression of Notch1 activity in cancer cells, we should individually create the treatment for the specific patient. Evidently, one patient would need a low dose of a two-drug mixture, while another patient would receive a high dose of the mixture to suppress the cancer activity. This may explain the observed differences in response to various patients with the same cancer to the anti-proliferative therapy. 

In the present study, we also observed that altered Notch1 activity in TNBC–related MDA-MB-231 cells results in the increased sensitivity of these cells for CDDP treatment, proving that measuring the prognostic value of Notch1 expression can help to guide individual therapy for BC patients, as suggested in other reports [35].

Recently, direct involvement of histone acetyltransferase (HAT) p300 and HDAC sirtuin 1 (SIRT1) in Notch signaling has been postulated—both enzymes are supposed to acetylate the intracellular domain of Notch receptor (NICD), thereby modulating signaling strength [53]. Therefore, HDIs could be regarded as new compounds for Notch-targeted therapy for cancer cells. Despite the accessibility of efficacious Notch inhibitors such as γ-secretase inhibitors (GSIs), peptides, antibodies, or probodies, Notch-related therapy is currently limited by serious adverse effects [54]. As we demonstrated previously, combined CDDP/HDIs treatment does not significantly affect normal cells [42], therefore, these drug combinations could be considered as a potential therapeutic tool for breast cancers. 

## 4. Materials and Methods 

### 4.1. Drugs

Cisplatin (CDDP) and valproic acid (VPA) were purchased from Sigma (St. Louis, MO, USA) and dissolved in phosphate buffered saline (PBS) with Mg^2+^ and Ca^2+^ at 1 mg/mL and 100 mM concentration as stock solutions, respectively. Suberoylanilide hydroxamic acid (SAHA) was purchased from Cayman Chemical (San Diego, CA, USA) and was prepared in dimethyl sulfoxide (DMSO) at 10 mM concentration as a stock solution. The reagents were diluted in order to obtain the final concentration with respective culture medium.

### 4.2. Cell Lines 

MDA-MB-231 (ATTC©HTB-26TB) breast cancer cell line was obtained from the American Type Culture Collection (Manassas, VA, USA). Breast cancer cells were grown in DMEM/HAM F12 culture medium (Sigma, St. Louis, MO, USA) supplemented with 10% fetal bovine serum (FBS) (Sigma), 100 IU/mL of penicillin (Sigma), and 100 µg/mL of streptomycin (Sigma). Mycoplasma free cultures were maintained at 37 °C in a humidified atmosphere with 5% CO_2_.

### 4.3. Transfection Procedure and Development of Breast Cancer Cell Lines with Decreased (Notch1^low^) and Increased (Notch1^high^) Notch1 Activity

MDA-MB-231 breast cancer cells were transfected with Lipofectamine 3000 Transfection Reagent (Thermo Scientific, Rockford, IL, USA) with plasmids carrying the N1ICD (Notch1 intracellular domain) or dnCSL (dominant negative CSL) according to the manufacturer’s protocol. 1 × 10^5^ cells were plated in a 24-well plate (80% of confluency). The next day, 1 µg of plasmid DNA was mixed with 50 µL of Opti-MEM medium and 2 µL of P3000 Reagent. Then, diluted DNA was added to diluted Lipofectamine 3000 Reagent (1:1), incubated for 15 min at room temperature (RT) and added to the cells. Cells were incubated for four days at 37 °C and then selected with puromycin (3 μg/mL).

### 4.4. Protein Extraction and Western Blotting Analysis

MDA-MB-231, Notch1^low^MDA-MB-231, and Notch1^high^MDA-MB-231 breast cancer cells (2.5 × 10^5^ cells/mL) were cultured for 24 h in 6-well plates (Nunc, Rochester, NY, USA). The cells were washed with PBS and lysed in RIPA buffer (ready-to-use solution containing 150 mM NaCl, 1.0% IGEPAL^®^ CA-630, 0.5% sodium deoxycholate, 0.1% SDS, 50 mM Tris, pH 8.0) (Sigma) enriched with protease inhibitor cocktail (Sigma) for 1 h at 4 °C. Protein concentration was quantified using a BCA protein assay kit (Pierce^®^ BCA Protein Assay Kit, Thermo Scientific). For Western blot analysis, supernatants of RIPA cell lysates were solubilized in 6 x Laemmli Sample Buffer (50% glycerol, 10% SDS, 300 mM Tris-HCl pH 6.8, 0.05% bromophenol blue, 6.25% β-mercaptoethanol) and denaturated for 5 min at 100 °C. 20 µg of protein extracts were loaded on 10% SDS polyacrylamide gel (SDS-PAGE) and separated electrophoretically. The proteins were transferred onto the Immobilon P membrane (Merck, Darmstadt, Germany). Following the transfer, the membrane was blocked with blocking solution (5% non-fat dried milk in TBS/0.1% Tween-20 (TBST)) for 1 h at RT and incubated overnight at 4 °C with the following primary antibodies: Anti-Notch1 (1:1000 in 5% non-fat dried milk/TBST, mouse monoclonal, Santa Cruz, Dallas, TX, USA), anti-β-actin (1:500 in 5% non-fat dried milk/TBST, mouse monoclonal, Santa Cruz). β-actin was used as a load control. On the following day, the membrane was washed and then incubated with an appropriate horseradish peroxidase-labeled secondary antibody (1:250 in 5% non-fat dried milk/TBST, Santa Cruz) for 1 h at RT. Finally, the proteins on the membrane were visualized using a Lumi-Light Western Blotting Substrate (Roche, Fishers, IN, USA) according to the manufacturer’s protocol.

### 4.5. Luciferase Reporter Assay

MDA-MB-231, Notch1^low^MDA-MB-231, and Notch1^high^MDA-MB-231 breast cancer cells were transfected with 12xCSL-Luc and CMV-LacZ plasmids using Lipofectamine 3000 (Thermo Fisher Scientific, Waltham, MA, USA) according to the manufacturer’s protocol. After 48 h, the cells were lysed in Cell Culture Lysis Reagent from Promega (Madison, WI, USA) and analyzed for luciferase activity with Luciferase assay system (Promega) using an Infinite M200 Pro microplate reader (Tecan, Männedorf, Switzerland) according to the protocols.

### 4.6. RNA Isolation and cDNA Synthesis

MDA-MB-231 breast cancer cells were seeded into 6-well culture plates at a density of 2.5 × 10^5^ cells/mL. The next day, the cells were incubated with selected concentrations of VPA or SAHA separately or in combination with CDDP for 24 h. Total RNA from the cells was isolated using the Extractme Total RNA Isolation Kit (Blirt, Gdansk, Poland) following the manufacturer’s instruction. The RNA concentration was determined using NanoQuant Plate and Tecan Infinite M200 Pro (Männedorf, Switzerland) at 260/280 nm. 1 μg of total RNA was reverse transcribed using the High Capacity cDNA Reverse Transcription Kit (Thermo Fisher Scientific, Waltham, MA, USA) according to manufacturer’s protocol.

### 4.7. Quantitative PCR (qPCR)

Quantitative PCR (qPCR) was performed using LightCycler^®^480 II instrument (Roche) in a mixture containing PowerUp SYBR Green Master Mix (Applied Biosystem, Foster City, CA, USA), 10 ng of cDNA and specific primers in a total volume of 10 µl. The gene-specific oligonucleotide primer sequences used in the present study were as follows: Notch1 (For: 5′-CAACTGCCAGAACCTTGTGC-3′, Rev: 5′-GGCAACGTCAACACCTTGTC-3′) and GAPD (For: 5′-CTCTGCTCCTCCTGTTCGAC-3′, Rev: 5′-GCCCAATACGACCAAATCC-3′). Relative quantification of gene expression was calculated based on the comparative C_T_ (threshold cycle value) method (ΔC_T_ = C_T_ gene of interest—C_T_ housekeeping gene). 

### 4.8. Cell Viability Assay

MDA-MB-231 [28], Notch1^low^MDA-MB-231, and Notch1^high^MDA-MB-231 breast cancer cells were platted on 96-well microplates at a density of 3 × 10^4^ cells/mL. The cells were incubated with CDDP (0.01–10 μg/mL), VPA (10–1000 μg/mL), or SAHA (0.02–3 μg/mL) for 96 h. Then, the cells were incubated with the MTT [3-(4,5-dimethylthiazol-2-yl)-2,5-diphenyltetrazolium bromide] solution (5 mg/mL, Sigma) for 3 h. During this time, MTT was metabolized by living cells to purple formazan crystals, which were solubilized in a sodium dodecyl sulfate (SDS) buffer (10% SDS in 0.01 N HCl) overnight. The optical density of the product was measured at 570 nm using an Infinite M200 Pro microplate reader (Tecan, Männedorf, Switzerland). The results of combined treatment of CDDP and HDIs were analyzed according to the isobolographic protocol. The drug doses were determined based on the IC_50_ values.

### 4.9. Isobolographic Analysis of Pharmacological Interactions between HDIs and CDDP

Pharmacological interactions between drugs for various cancer cell lines were analyzed using the isobolographic analysis, as described previously [28,41]. To begin isobolographic analysis of interaction between CDDP and SAHA or VPA, we determined the inhibition of cell viability of Notch1^low^MDA-MB-231 and Notch1^high^MDA-MB-231 breast cancer cell lines. From log-probit dose-response effects of CDDP, SAHA, and VPA in two cancer cell lines, we calculated median inhibitory concentrations (IC_50_ values) for the tested compounds, as advised earlier [28]. As the dose-response effects for CDDP, SAHA, and VPA in all the investigated cell lines were non-parallel to one another, a type I isobolographic analysis for non-parallel dose-response effect curves was used, as advised earlier [28]. The type of interactions between CDDP and SAHA or VPA was established by comparing the experimentally determined IC_50_ mix values (at the fixed-ratio of 1:1) with the theoretically calculated additive IC_50_ add values, according to the methods described elsewhere [28,39,41]. The isobolographic analysis permits accurate classification of the observed interactions of drugs used in the mixture at the fixed drug dose ratio (mostly, 1:1). Theoretically, four types of interaction can be discerned: Supra-additivity (synergy), additivity, sub-additivity (relative antagonism), and infra-additivity (absolute antagonism) [28]. 

### 4.10. Statistical Analysis

The data was analyzed using GraphPad Prism software (San Diego, CA, USA) with one-way ANOVA and Tukey post-hoc testing. Results were presented as mean ± standard error of the mean (± S.E.M.). *p* < 0.05 was considered to indicate a statistically significant difference. Log-probit analysis was used to determine the experimentally derived IC_50_ and IC_50_ mix values for CDDP, SAHA, and VPA, when the drugs were administered alone or in combination for the fixed ratio of 1:1. Statistical difference between the experimentally-derived IC_50_ mix values and the theoretically calculated additive IC_50_ add values (for lower and upper line of additivity) was assessed with unpaired Student’s t-test, as presented elsewhere [28]. 

## Figures and Tables

**Table 1:** IC_50_ values (µg/mL) for CDDP and HDIs (SAHA and VPA) in transfected and native [28] MDA-MB-231 breast cancer cells.

Cell Line	CDDP	SAHA	VPA
Notch1^high^MDA-MB-231	0.265	1.027	638.5
MDA-MB-231	3.614	0.577	267.0
Notch1^low^MDA-MB-231	0.130	0.890	628.5

**Table 2:** Type I isobolographic analysis of interactions (for non-parallel log-probit dose–response relationship curves (DRRCs) between CDDP and SAHA or VPA at the fixed-ratio combination of 1:1 in MDA-MB-231 with increased (Notch1^high^MDA-MB-231) or decreased (Notch1^low^MDA-MB-231) activity of Notch1 measured in vitro by the MTT assay. Results are presented as median inhibitory concentrations (IC_50_ values in μg/mL ± S.E.M.) for two-drug mixtures, determined either experimentally (IC_50_ mix) or theoretically calculated (IC_50_ add) from the equations of additivity (Tallarida 2006, 2007), blocking proliferation in 50% of tested cells in cancer cell lines (MDA-MB-231 with increased or decreased activity of Notch1) measured in vitro by the MTT assay. n mix—total number of items used at those concentrations whose expected anti-proliferative effects ranged between 16% and 84% (i.e., 4 and 6 probits) for the experimental mixture; n add—total number of items calculated for the additive mixture of the drugs examined; L IC_50_ add value calculated from the equation for the lower line of additivity; U IC_50_ add value calculated from the equation for the upper line of additivity. Statistical evaluation of data was performed with an unpaired Student’s t-test.

Cell Line	Notch1 Activity	Combination	IC_50_ mix (μg/mL)	n_mix_	L IC_50_ add (μg/mL)	n_add_	U IC_50_ Add (μg/mL)	n_add_
Notch1^high^MDA-MB-231	Increased	CDDP+SAHA	0.382 ± 0.137	24	0.392 ± 0.253	50	0.902 ± 0.244	50
Notch1^high^MDA-MB-231	Increased	CDDP+VPA	286.9 ± 193.2	30	303.5 ± 255.7	56	336.0 ± 272.0	56
Notch1^low^MDA-MB-231	Decreased	CDDP+SAHA	0.903 ± 0.407	30	0.391 ± 0.279	50	0.629 ± 0.310	50
Notch1^low^MDA-MB-231	Decreased	CDDP+VPA	381.5 ± 192.9	30	289.7 ± 214.5	56	392.8 ± 246.0	56

**Table 3:** Types of interactions between CDDP and SAHA or VPA at the fixed-ratio combination of 1:1 in MDA-MB-231 cancer cell lines with increased (Notch1^high^MDA-MB-231) or decreased (Notch1^low^MDA-MB-231) activity of Notch1 with reference to MDA-MB-231 breast cancer cells with native level of Notch1 activity (MDA-MB-231^native^) [28] measured in vitro by the MTT assay.

Combination	Notch1^high^MDA-MB-231	Notch1^low^MDA-MB-231	MDA-MB-231^native^ [28]
CDDP/VPA	additivity	additivity	antagonism
CDDP/SAHA	additivity	additivity with tendency towards antagonism	additivity

## Abbreviations

ANOVA	Analysis of variance
BC	Breast cancer
CBF1	Centromere-binding protein 1
CDDP	Cisplatin
CSL	CBF1/Su(H)/Lag-1
DCIs	Ductal carcinoma in situ
DRRCs	Log-probit dose–response relationship curves
Dll	Delta like ligand
DMSO	Dimethyl sulfoxide
DNA	Deoxyribonucleic acid
dnCSL	Dominant negative CSL
DSL	Delta, Serrate, Lag2
ER	Estrogen receptor
FBS	Fetal bovine serum
GSIs	γ-secretase inhibitors
HAT	Histone acetyltransferase
HDIs	Histone deacetylase inhibitors
HER2	Human epidermal growth factor receptor 2
HES1	HES family bHLH transcription factor 1
IDCs	Invasive ductal carcinomas
IHC	Immunohistochemistry
ILCs	Invasive lobular carcinomas
KDM5A	Lysine-specific demethylase 5A
MTT	3-(4,5-dimethylthiazol-2-yl)-2,5-diphenyltetrazolium bromide
NICD	Intracellular domain of Notch receptor
PCAF	Acetyltransferase p300/CBP associated factor
PBS	Phosphate buffered saline
PR	Progesteron receptor
RBP-Jk	Recombination signal binding protein for immunoglobulin kappa
SAHA	Suberoylanilide hydroxamic acid
SHARP	SMART/HDAC1-associated repressor protein
SCLC	Small-cell lung carcinoma
SDS	Sodium dodecyl sulfate
S.E.M.	Standard error
SIRT1	Sirtuin 1
TNBC	Triple negative breast cancer
VPA	Valproic acid
